# Sodium vanadium titanium phosphate electrode for symmetric sodium-ion batteries with high power and long lifespan

**DOI:** 10.1038/ncomms15888

**Published:** 2017-06-29

**Authors:** Dongxue Wang, Xiaofei Bie, Qiang Fu, Ditty Dixon, Natalia Bramnik, Yong-Sheng Hu, Francois Fauth, Yingjin Wei, Helmut Ehrenberg, Gang Chen, Fei Du

**Affiliations:** 1 Key Laboratory of Physics and Technology for Advanced Batteries (Ministry of Education), State Key Laboratory of Superhard Materials, College of Physics, Jilin University, Changchun 130012, China; 2 Institute for Applied Materials (IAM), Karlsruhe Institute of Technology (KIT), D-76344 Eggenstein-Leopoldshafen, Germany; 3 Key Laboratory for Renewable Energy, Beijing Key Laboratory for New Energy Materials and Devices, Beijing National Laboratory for Condensed Matter Physics, Institute of Physics, Chinese Academy of Sciences, School of Physical Sciences, University of Chinese Academy of Sciences, Beijing 100190, China; 4 CELLS-ALBA Synchrotron, Cerdanyola del Valles, E-08290 Barcelona, Spain

## Abstract

Sodium-ion batteries operating at ambient temperature hold great promise for use in grid energy storage owing to their significant cost advantages. However, challenges remain in the development of suitable electrode materials to enable long lifespan and high rate capability. Here we report a sodium super-ionic conductor structured electrode, sodium vanadium titanium phosphate, which delivers a high specific capacity of 147 mA h g^−1^ at a rate of 0.1 C and excellent capacity retentions at high rates. A symmetric sodium-ion full cell demonstrates a superior rate capability with a specific capacity of about 49 mA h g^−1^ at 20 C rate and ultralong lifetime over 10,000 cycles. Furthermore, *in situ* synchrotron diffraction and X-ray absorption spectroscopy measurement are carried out to unravel the underlying sodium storage mechanism and charge compensation behaviour. Our results suggest the potential application of symmetric batteries for electrochemical energy storage given the superior rate capability and long cycle life.

## Abstract (web summary)

Discovering suitable electrodes is a challenge for the development of sodium-ion batteries. Here the authors demonstrate a high-performance symmetric battery based on Na_2_VTi(PO_4_)_3_, highlighting its potential application in a smart grid.

With increasing demands for renewable energy sources, wind and solar energies are taking on a more important role in the global energy mix. The inherently variable nature of their electricity generation, however, brings additional challenges to already stressed power grids that must match power generation and provide optimum customer service at any time. To meet future energy storage needs, the breakthroughs in the advanced battery technologies are urgently demanded^1^,^2^. In the recent years, sodium-ion batteries (SIBs) have attracted particular interest as one of the most promising solutions to grid energy storage because of the low cost and abundant resources of sodium salts in the Earth’s crust and oceans, in sharp contrast to the limited resources and uneven distribution of lithium^3^,^4^,^5^. Moreover, SIBs shares the identical ‘rocking-chair’ mechanism with lithium-ion batteries, as well as the similar chemical and physical properties between sodium and lithium, which is beneficial for the development of SIBs following the successful pathway of lithium-ion batteries.

Among the numerous identified electrode materials, a series of phosphates with a sodium super-ionic conductor (NASICON) structure are particularly attractive considering that their stable crystallographic structure could enable long-term cycling and improved safety. As shown in Fig. 1a, NASICON-structured phosphates Na_2_MM′(PO_4_)_3_ are constructed by the ‘lantern-like’ units sequenced along the *c* axis, which are composed by two [MO_6_]/[M′O_6_] octahedra and three [PO_4_] tetrahedra by sharing all their corners^6^. This robust framework provides a three-dimensional diffusion pathway for Na^+^ ions with controllable lattice expansion below 8% (refs 7, 8), which is believed beneficial for the cycling stability. Furthermore, the strong inductive effect of polyanion allows to monitor the M^*n*+^/M^(*n*−1)+^ redox pair and gives rise to higher working potential versus Na^+^/Na than in oxides. More importantly, a flexible atomic arrangement is adopted with an extreme versatility towards cation substitution for a given structure, favourable for materials optimization and design. So far, some phosphates have been synthesized and reported to deliver considerable sodium storage performance as either cathode (for example, Na_3_V_2_(PO_4_)_3_ (refs 9, 10), Na_3_V_2_O_2*x*_(PO_4_)_2_F_3−2*x*_ (refs 11, 12) and so on) or anode (for example, NaTi_2_(PO_4_)_3_ (refs 13, 14), Na_3_Ti_2_(PO_4_)_3_ (ref. 15) and so on).

Recently, symmetric SIBs full cell have been designed to exploit the voltage differences between distinct redox couples, such as P2-type Na_0.6_[Cr_0.6_Ti_0.4_]O_2_ (ref. 16), O3-type Na_0.8_Ni_0.4_Ti_0.6_O_2_ (ref. 17) and Na_3_V_2_(PO_4_)_3_ (ref. 18). Compared to the non-symmetrical cells employing alloying-type materials^19^,^20^ or hard carbon^21^,^22^ as anodes, the symmetric cells based on the same intercalation-type compound could greatly suppress the volume expansion and the safety concern of sodium dendrites at low working potentials. Also from a technical point of view, the assembly of symmetric cell could simplify the fabrication processes and significantly reduce the manufacturing costs. However, it should be noted that the high rate capability of the symmetric cell, as well as the lifespan, should be further improved to demonstrate the efficacy for commercial applications. NASICON-structured Na_2_VTi(PO_4_)_3_ has been prepared recently and its electrochemical property is once evaluated in the aqueous system^23^. However, the working voltage is limited due to the redox limitation of aqueous system with only one Na^+^ ion per formula insertion. Its crystal structural and sodium storage property in the organic electrolyte system have not been studied.

Here in this work, Na_2_VTi(PO_4_)_3_ is synthesized with a facile sol-gel method and shows a stable specific capacity of about 147 mA h g^−1^ between 4.5 and 1.5 V versus Na^+^/Na with a coulombic efficiency of over 99%, far higher than that in aqueous electrolyte. By taking advantage of the distinct redox couples between V^3+^/V^4+^ and Ti^4+^/Ti^3+^, the assembled full cell using Na_2_VTi(PO_4_)_3_ as both cathode and anode achieves high-rate charge–discharge at a high rate of 20 C (the current density of 2.5 A g^−1^) and ultralong lifespan over 10,000 cycles, which is not attainable in the state-of-the-art SIBs. Moreover, aided by *in situ* synchrotron and X-ray absorption spectrum, we are able to identify a two-phase reaction mechanism followed by a solid-solution process, in addition to the stepwise redox behaviour during the initial sodiation/desodiation reactions.

## Results

### Crystal structure of Na_2_VTi(PO_4_)_3_ and characterization

**Figure 1:**
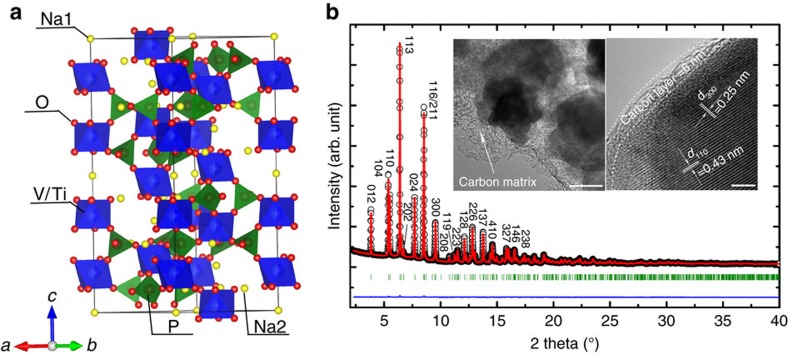
Crystal structure of Na_2_VTi(PO_4_)_3_. (**a**) Schematic crystal structure of Na_2_VTi(PO_4_)_3_, and *a*–*c* represent different axes. (**b**) Rietveld refinement based on the synchrotron diffraction data. Black circle, red line and blue line represent the observed, calculated and difference patterns, respectively. The olive tick marks correspond to the Bragg reflections; the inset shows the TEM (scale bar, 100 nm) and HRTEM images (scale bar, 4 nm).

The powder synchrotron X-ray diffraction pattern of Na_2_VTi(PO_4_)_3_, as shown in Fig. 1b, can be indexed assuming a rhombohedral unit cell of *a*=8.5992(1) Å and *c*=21.8181(4) Å. The similar lattice parameters to those of Na_3_V_2_(PO_4_)_3_ (refs 7, 9) and NaTi_2_(PO_4_)_3_ (refs 14, 24) and the fact that no specific diffraction lines are extinct indicate that the current phase is isostructural to these phases and crystallizes also in the space group *R*
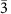
*c*. The Rietveld refinement based on the synchrotron diffraction data converged to *R*_wp_=4.39% and *R*_p_=3.22% with reasonable displacement and site occupancy factors for all atoms as listed in Supplementary Table 1, suggesting a reliable structural analysis. It should be noted several extra peaks can be identified between 4^o^ and 10^o^ in the enlarged diffraction pattern, see Supplementary Fig. 1. The corresponding impurity phase(s) are not readily visible as a result of their low amount and are considered as not relevant for the following electrochemical studies. As illustrated in Fig. 1a, the crystal structure consists of corner-sharing [TiO_6_]/[VO_6_] octahedra and [PO_4_] tetrahedra. An octahedral 12*c* site is randomly occupied by V^3+^ and Ti^4+^ ions. Transmission electron microscopy (TEM) confirms the compositing manner between Na_2_VTi(PO_4_)_3_ particles and amorphous carbon. As displayed in the inset of Fig. 1b, well-crystallized Na_2_VTi(PO_4_)_3_ nanoparticles of ca. 200 nm in size are dispersed within the amorphous carbon matrix. The TEM image also reveals that a thin layer of carbon of ca. 6 nm in thickness is coated on the surface of Na_2_VTi(PO_4_)_3_ particles. The lattice fringes of 0.43 and 0.25 nm can be observed inside the nanoparticles, consistent with the interplanar spacing of (110) and (300) planes, respectively, for the rhombohedral Na_2_VTi(PO_4_)_3_. Element mapping (Supplementary Fig. 2) of the selected area indicates a homogeneous distribution of various elements in the pristine material, including amorphous carbons. Qualitatively, the degree of graphitization is studied by Raman spectroscopy (Supplementary Fig. 3), which suggests the amorphous nature of the compositing carbon in the pristine Na_2_VTi(PO_4_)_3_@C hybrid since *I*_D_/*I*_G_=0.98 (ref. 25). The amount of carbon is estimated to be about 4.2 wt% according to the carbon-hydrogen-nitrogen (CHN) element analysis. This optimal architectural design with carbon-coated Na_2_VTi(PO_4_)_3_ nanoparticles embedded in amorphous carbon framework could overcome the disadvantage of low electronic conductivity of NASIONC-type materials and facilitate fast electron and ionic transfer.

### Sodium storage performance

**Figure 2:**
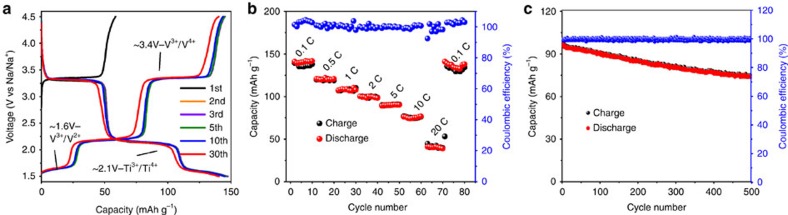
Sodium storage performance of Na_2_VTi(PO_4_)_3_@C electrode. (**a**) The 1st, 2nd, 3rd, 5th, 10th and 30th galvanostatic charge–discharge profiles between 1.5 and 4.5 V at a current density of 0.1 C (1 C corresponds to 125 mA g^−1^). (**b**) Rate capability from 0.1 to 20 C. (**c**) Long-term cycle life over 500 cycles at 10 C rate.

The electrochemical properties of Na_2_VTi(PO_4_)_3_@C hybrid were examined in a CR2032 half-cell with metallic sodium as the counter electrode. As displayed in Fig. 2a, the galvanostatic charge–discharge profiles were recorded between 1.5 and 4.5 V at the current density of 0.1 C (1 C corresponds to 125 mA g^−1^). In the initial charging process, the hybrid electrode exhibits a working potential plateau at around 3.4 V versus Na^+^/Na, similar to the initial charging behaviour of Na_3_V_2_(PO_4_)_3_, which could be attributed to the oxidation of V^3+^ into V^4+^ (ref. 8). In contrast, its initial discharging profile is featured by a three-step transitions at 3.4, 2.1 and 1.6 V resulting from the stepwise reduction processes of V^4+^→V^3+^ (ref. 9), Ti^4+^→Ti^3+^ (ref. 24) and V^3+^→V^2+^ (ref. 26), respectively, which will be discussed in detail together with *in situ* X-ray absorption near-edge structure. The initial charge capacity attained is about 59 mA h g^−1^, corresponding to the extraction of nearly 0.95 Na^+^ ions. The discharge capacity is 147 mA h g^−1^ when initially discharged to 1.5 V, indicative of 2.4 Na^+^ ions inserted. After the second cycle, all the voltage-capacity profiles are analogous, suggesting an excellent reversibility during repeated Na-ion cycling. Na_2_VTi(PO_4_)_3_@C hybrid also exhibits good cycle performance with a capacity retention of 96% over 30 cycles at 0.1 C and a highly stable coulombic efficiency of nearly 100% (Supplementary Fig. 4). Figure 2b shows the charge–discharge capability of the hybrid electrode at progressively increased rates, with reversible capacities of 140, 120, 107, 100, 90, 77 and 44 mA h g^−1^ as the current rate increased from 0.1, 0.5, 1, 2, 5, 10 to 20 C rates, respectively. When the applied current rate is returned to 0.1 C after 70 cycles, Na_2_VTi(PO_4_)_3_@C delivers a discharge capacity of 140 mA h g^−1^, which demonstrates a superior rate capability again. The voltage-capacity profiles at various current densities are displayed in Supplementary Fig. 5. As shown, enhanced polarization of the cell occurs during the charge and discharge profiles as current density is increased. Even so, the voltage plateaus, characteristic of V^4+^/V^3+^ and Ti^4+^/Ti^3+^ redox pairs, are retained at a rate of 10 C, suggesting that the dominance of insertion/extraction processes. Inspired by the excellent rate capability, the long-term cycle stability is also tested at 10 C, as displayed in Fig. 2c. Under these conditions, the capacity retention is 77% after 500 cycles and the coulombic efficiency was nearly 100%.

**Figure 3:**
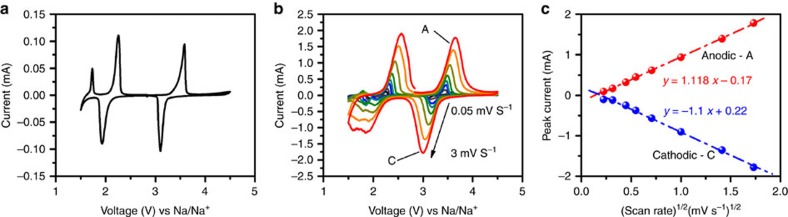
Kinetics properties of Na_2_VTi(PO_4_)_3_@C electrode. (**a**) Cyclic voltammogram curve of the Na_2_VTi(PO_4_)_3_@C nanocomposite at a scan rate of 0.05 mV s^−1^ between 1.5 and 4.5 V. (**b**) Cyclic voltammograms curves at different scanning rates. (**c**) The relationship between the peak current (*I*_p_) and the square root of the scan rate (*ν*^1/2^).

To further examine the redox activities of Na_2_VTi(PO_4_)_3_@C electrode, the cyclic voltammetry (CV) curve for the initial cycle is recorded at the sweep rate of 0.05 mV s^−1^ in the potential region of 1.5−4.5 V versus Na^+^/Na, as shown in Fig. 3a. Three pairs of redox couples are observed, consistent with the working plateaus found in the voltage-capacity profiles. The kinetic behaviour is further studied utilizing the CV analysis with different voltage scanning rates (Fig. 3b). Fitting the linear relationship between the peak currents and the square root of the scanning rate for A and C peaks (Fig. 3c) yields comparable Na^+^ ion apparent diffusion coefficient *D*_Na+_ values (2.12 × 10^−10^ and 2.19 × 10^−10 ^cm^2^ s^−1^ for A and C, respectively) to those for the NASICON-structured Na_3_V_2_(PO_4_)_3_/C nanocomposites^9^,^27^ and several layered cathode materials^28^,^29^ used in SIBs, according to the Randles−Sevick equation^9^:


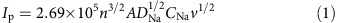


where *I*_p_ is the peak current of anodic or cathodic peaks, *n* is the number of electron per formula during the insertion, *A* is the effective contact area between the electrode and electrolyte with a commonly used value of 0.64 cm^2^, *C*_Na_ is the concentration of Na^+^ in the electrode, and *D*_Na+_ is the diffusion coefficient of Na^+^ and *v* is the scan rate.

**Figure 4:**
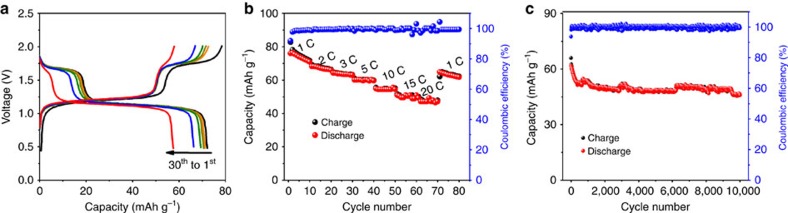
Electrochemical performance of the symmetric full cell. (**a**) Galvanostatic charge–discharge profiles between 0.5 and 2.0 V at a current density of 1 C. (**b**) Rate capability from 1 to 20 C. (**c**) Long-term cycle life over 10,000 cycles at a current rate of 10 C.

Taking advantage of the voltage difference between V^3+^/V^4+^ and Ti^4+^/Ti^3+^ redox couples, a symmetric SIBs full cell is assembled configured [Na_2_VTi(PO_4_)_3_@C∥1 M NaClO_4_ in (EC:PC=1:1)∥Na_2_VTi(PO_4_)_3_@C]. The voltage-capacity profiles of this symmetric cell at 1 C (the battery is anode limited) are shown in Fig. 4a. The initial discharge and charge capacities were 78 mA h g^−1^ and 72 mA h g^−1^, respectively, with the high initial coulombic efficiency of 92%. The good capacity retention is obtained as 81% over 30 cycles (Supplementary Fig. 6). CV profiles at a scanning rate of 0.1 mV s^−1^ between 2.0 and 0.5 V (Supplementary Fig. 7) show two symmetric redox pairs at ca. 1.7 and 1.2 V, consistent with its charge–discharge behaviour. The low voltage pair is originated from the voltage difference between 3.4 V (V^3+^/V^4+^)^9^ and 2.1 V (Ti^4+^/Ti^3+^)^10^,^24^ while, the higher pair is mainly due to a small portion of contributions from V^3+^ into V^2+^ during the sodiation process^26^. Note that the working voltage is lower than those based on Na_3_V_2_(PO_4_)_3_ or layered compounds, which might result in the lower energy density. Encouragingly, the symmetric cell based on Na_2_VTi(PO_4_)_3_@C electrodes can realize a high-rate charge–discharge at 20 C with a specific capacity of 49 mA h g^−1^ (Fig. 4b) and an ultralong lifespan of this symmetric cell is achieved over 10,000 cycles with capacity retention of 74% at 10 C (Fig. 4c), which is critically important for their practical application to smooth the intermittency of renewable energies and integrate them into the grid. It should be noticed that, as compared in Supplementary Table 2, the rate performance and cycle stability are among one of the best performance characteristics for the state-of-the-art symmetric^15^,^16^,^17^,^18^,^30^,^31^,^32^,^33^ and non-symmetric SIBs full cells^13^,^19^,^20^,^21^,^22^,^34^,^35^. The excellent sodium storage performance might be strongly related to the symmetric character of the full cell, which serves to suppress the volume expansion during Na^+^ reversible insertion/extraction. Moreover, the intrinsic high ionic conductivity of NASICON-type compounds, as well as the improved electronic conductivity after composting with carbonaceous materials, will contribute to the superior rate capability.

## Discussion

**Figure 5:**
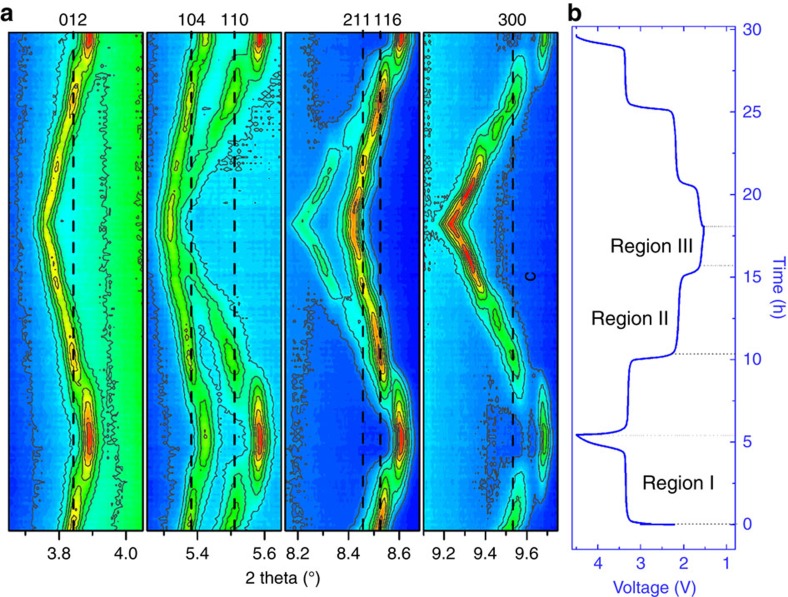
Structure evolution on Na^+^ extraction/insertion. (**a**) Contour maps of *in situ* synchrotron X-ray diffraction collected during the first charge–discharge and subsequent charge process of the Na/Na_2_VTi(PO_4_)_3_@carbon cell. (**b**) The corresponding charge–discharge profiles at a current rate of C/10 in a voltage range between 1.5 and 4.5 V.

To elucidate the structural evolution during the reversible sodium insertion and extraction, *in situ* synchrotron diffraction patterns for the initial charge–discharge and subsequent charge were collected in a voltage range of 1.5–4.5 V. Contour maps in the selected 2-theta ranges are shown in Fig. 5a. At the initial stage of charge, all reflections of Na_2_VTi(PO_4_)_3_ can be indexed to a rhombohedral cell with space group of *R*
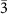
*c* and the refined parameters are listed in Supplementary Table 3. As charging proceeds, all peaks shift towards the high-angle side, indicative of the shrinkage of the lattice framework. On sodium extraction to 13 mAh g^−1^, a few extra diffraction lines appear at 3.9°, 5.6°, 8.6°, 9.7°, 12.2°, 12.9°, 13.9° and 14.8°. And, their intensities increase while those from the pristine electrode decrease and disappear on charging to 45 mAh g^−1^ (Region I). This leads to the notion of a phase transition from the parent phase Na_2_VTi(PO_4_)_3_ to a sodium poor phase Na_2–*x*_VTi(PO_4_)_3_. On a closer inspection on the diffraction line at 4.5 V by Rietveld refinement (Supplementary Fig. 8b and Supplementary Table 4), the new reflections could also be indexed to a rhombohedral symmetry with contracted lattice parameters. Refined *x* value in Na_*x*_VTi(PO_4_)_3_ is 0.923, well consistent with its initial charge capacity. Interestingly, in comparison with the unchanged occupancy of Na^+^ ions on the 6*b* sites during initial charge, that value for the 18*e* site becomes much smaller and close to zero, which might suggest that Na ions on the Na(2) sites have a weak bonding energy with their neighbouring atoms or polyanion groups and tend to be more easily extracted as a result of the loosened chemical environment. The volume shrinkage of Na_2_VTi(PO_4_)_3_ is estimated around 4% when initially charged to 4.5 V, which is smaller than that of Na_3_V_2_(PO_4_)_3_ (refs 7, 8) and Na_3_V_2_(PO_4_)_2_F_3_ (refs 36, 37). On initial discharging to nearly 2.4 V, the intensities of those new-born reflections in the initial charge, such as 3.9°, 5.6°, 8.6°, 9.7°, 12.2°, 12.9°, 13.9° and 14.8°, decrease. And, those belonging to the pristine Na_2_VTi(PO_4_)_3_, for example, 3.8°, 5.5°, 8.5°, 9.5°, 12.1°, 12.8°, 13.7° and 14.5°, reappear. All these features suggest a reversible phase transition occurs between 4.5 and 2.4 V.

When discharged from 2.4 V to nearly 1.9 V (Region II), a clear discrete transition is observed for the reflections of 5.5°, 8.4° and 9.5° at around 2.1 V, as presented in Fig. 5b. Furthermore, the evolution of diffraction lines at high-angle regions (Supplementary Fig. 9b) demonstrates the coexistence of two phases in this voltage region. All these features are well consistent with the plateau-like behaviour at 2.1 V in the charge–discharge profiles of Na_2_VTi(PO_4_)_3_ (Fig. 2a). This phase coexistence is in agreement with the necessarily discontinuous first-order transition between two iso-symmetric phases. In Region III (from 1.9 to 1.5 V), there is no observed formation of extra diffraction lines or disappearance of reflections, which can be viewed as a single-phase transition. Rietveld refinement based on the diffraction pattern at 1.5 V reveals the single phase with rhomobohedral symmetry (Supplementary Fig. 8c and Supplementary Table 5) and the Na^+^ content was refined to *x*=3.3(3), consistent with their theoretical discharge capacity at 1.5 V. It is important to note that, accompanied by the increasing occupancies at 18*e* Na2-site, the Na ions occupation on the 6*b* Na1-site decreases in comparison with the pristine sample and the one at 4.5 V. This might signify the movement of Na ions from the 6*b* to the 18*e* sites, or the interspace sites. In the subsequent charge from 1.5 to 4.5 V, Na_2_VTi(PO_4_)_3_ electrode demonstrates a highly reversible phase transition, which involves the stepped solid-solution and two two-phase reactions. Therefore, the excellent electrochemical performance of Na_2_VTi(PO_4_)_3_ can be attributed to the stable framework and the small reversible volume variation.

**Figure 6:**
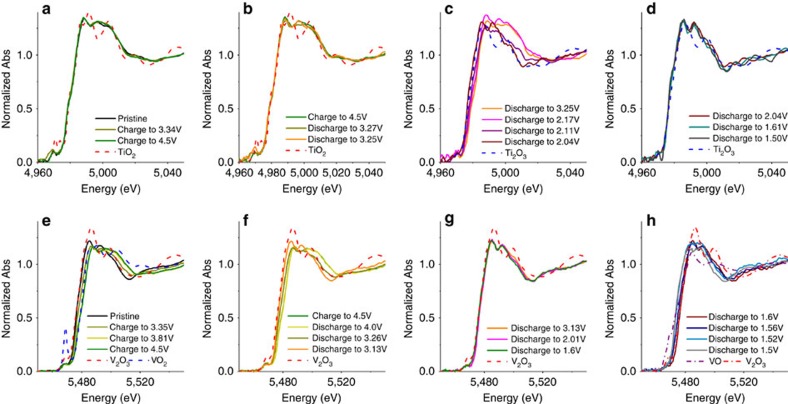
Charge compensation mechanism. *In situ* XANES for Ti K-edge (**a**–**d**) and V K-edge (**e**–**h**).

Moreover, *in situ* X-ray absorption near-edge structure (XANES) spectra (Fig. 6) are further collected on Na_2_VTi(PO_4_)_3_@C to look into the stepwise electron transfer behaviour. During the initial charge to 4.5 V, no significant changes in the edge energy position and shapes are observed in the Ti K-edge XANES spectra. It means the valence state of Ti is robust in this region. Compared to TiO_2_ reference (Fig. 6a), Ti is believed existing in its +4 oxidation state in the pristine state as well as in Region I. In contrast, the V K-edge spectra shifts towards the high-energy side during charging, indicative of an increasing oxidation state of V. On full charging (4.5 V), the spectrum overlaps with that of reference VO_2_ (Fig. 6e), confirming that the valence of V became +4. As a result, the main charge transfer during the initial charge can be attributed to the oxidation of V^3+^ to V^4+^. During the initial discharge till 3.1 V, the edge of V shifts to its lower energy. Moreover, the edge position of the spectrum corresponding to 3.1 V overlap with that of the reference V_2_O_3_, hinting the reversible reduction V^4+^ to V^3+^ (Fig. 6f). However, under the same discharge conditions, the Ti K-edge shows no shift, indicative of electrochemically inactive Ti in this potential window. With further discharge from ∼ 3.1 to 2.0 V, the edge region of Ti K-edge spectra shifts to lower energy and the one at 2.04 V almost reproduces the spectrum of Ti_2_O_3_. In contrast, the K-edge spectra for V do not shift. This means that the reduction of Ti^4+^ to Ti^3+^ plays a dominating role, in good agreement with the similar reduction potential of Ti^4+^/Ti^3+^ in Na_3_Ti_2_(PO_4_)_3_ (ref. 15). Below 2.0 V, a clear edge shift to lower energy is again observed for the V K-edge, in sharp contrast to the no significant move of Ti K-edge, indicating a further reduction of V^3+^ to its lower oxidation state in this potential regime. However, it should be noted that the XANES spectrum of V K-edge at 1.5 V could not fully match the one of the reference compound VO, possibly due to the difference in the coordination of VO_6_ octahedra between monoclinic VO and rhombohedral Na_2_VTi(PO_4_)_3_. Overall, in view of charge compensation behaviour, the charge and discharge process of Na_2_VTi(PO_4_)_3_ demonstrates a highly reversible characteristic.

In summary, by optimizing transition metal ions of V^3+^ and Ti^4+^ with similar ionic radii and a substantial difference in redox potential, we designed and synthesized a NASICON-structured compound Na_2_VTi(PO_4_)_3_. It is found that this material exhibits successive working plateaus at 3.4 and 2.1 V, corresponding to the redox couples of V^3+^/V^4+^ and Ti^4+^/Ti^3+^, respectively. By taking advantage of this potential difference, a symmetric full cell using hybrid Na_2_VTi(PO_4_)_3_@C electrodes is constructed, with manifested high-rate capacity and ultralong lifespan over 10,000 cycles. Moreover, *in situ* synchrotron and XAS measurements are carried out to unveil the structural evolution and charge compensation mechanism. Our study highlights the prospect of NASICON-type structure as a materials platform to improve the Na-ion storage performance for high-power and long-lived SIBs.

## Methods

### Preparation of Na_2_VTi(PO_4_)_3_@C nanocomposite

The NASICON-type Na_2_VTi(PO_4_)_3_@C nanocomposite was prepared by a sol-gel method. First, the stoichiometric amount of Na_2_CO_3_ (Sigma-Aldrich, 99.9%), NH_4_VO_3_ (Sigma-Aldrich, 99%), (CH_3_CH_3_CHO)_4_Ti (Sigma-Aldrich, 99%) and NH_4_H_2_PO_4_ (Sigma-Aldrich, 99%) with a molar ratio of 1:1:1:3 was dissolved in 0.02 M aqueous citric acid [HOC(COOH) (CH_2_–COOH)_2_] (Sigma-Aldrich, 99%) solution. The ratio of sodium:citric acid equals to 2:1. Then the solution was stirred at 80 °C for 12 h to get a precursor. The precursor was pretreated at 350 °C for 5 h, followed by sintering at 800 °C for 12 h under nitrogen atmosphere to obtain the Na_2_VTi(PO_4_)_3_@C nanocomposite.

### Characterization

Raman spectroscopy was performed on a Renishaw inVia Raman microscope with Ar-ion laser excitation (*λ*=514.5 nm). The content of carbon in the Na_2_VTi(PO_4_)_3_@C nanocomposite was evaluated using a Mettler-Toledo CHN analyzer. A FEI Tecnai G2-type TEM was used to investigate morphology and microstructure. Synchrotron diffraction was performed at the powder diffraction beam line (MSPD) at ALBA, Barcelona, using synchrotron radiation with an energy of 30 KeV (*λ*=0.413364 Å) and a MYTHEN 1D position-sensitive detector. *In situ* XAS measurements were carried out at the P65 beamline at PETRA III (DESY, Hamburg). XAS spectra where recorded in quick-XAS (10 min per spectrum) mode in fluorescence geometry using a PIPS diode. Both Ti as well as V K-edges where measured in one go. All the XAS spectra were processed using DEMETER software package.

### Electrochemical measurement

The electrochemical properties of the as-prepared Na_2_VTi(PO_4_)_3_@C nanocomposite were examined by assembling coin-type half cells with sodium foil as the counter electrode. The electrodes were prepared by coating a slurry, in which the Na_2_VTi(PO_4_)_3_@C active materials, super P conductive and polyvinylidene fluoride binders dissolved in *N*-methylpyrrolidone (NMP) were mixed in a weight ratio of 7:2:1 on an aluminium foil current collector. The cathode loading mass is around 1.75 to 2.2 mg cm^−2^. These electrode films were dried in a vacuum oven at 120 °C for 10 h. After dividing the electrode film into square parts of 0.8 × 0.8 cm^2^, coin cells were assembled in a glove box. The separators of the cells were a glass fibre filter (Whatman GF/C). The electrolyte was 1 M NaClO_4_ dissolved in a solvent of ethylene carbonate (EC) and propylene carbonate (PC) (1:1 v/v). Galvanostatic charge–discharge cycling was then performed on a Land-2001A (Wuhan, China) automatic battery tester. CV was performed on a VSP multichannel potentiostatic–galvanostatic system (Bio-Logic SAS, France). In symmetrical sodium-ion battery test, the battery was assembled into a coin-type full cell and was anode limited with as-prepared Na_2_VTi(PO_4_)_3_@C as both cathode and anode. Cell balance was achieved by setting the electrode mass ratio of cathode/anode to 1.6 (the anode loading mass is around 1.75–2.2 mg cm^−2^). 1 C corresponds to 125 mA g^−1^.

### Data availability

Data supporting the findings of this study are available within the article and its Supplementary Information and from the corresponding author on reasonable request.

## Additional information

**How to cite this article:** Wang, D. *et al*. Sodium vanadium titanium phosphate electrode for symmetric sodium-ion batteries with high power and long lifespan. *Nat. Commun.* **8,** 15888 doi: 10.1038/ncomms15888 (2017).

**Publisher’s note:** Springer Nature remains neutral with regard to jurisdictional claims in published maps and institutional affiliations.

## Supplementary Material

Supplementary Information Supplementary Figures and Supplementary Tables
