# Supplementary material for: Sodium vanadium titanium phosphate electrode for symmetric sodium-ion batteries with high power and long lifespan
Source: Nat Commun. 2017 Jun 29;8:15888. doi: 10.1038/ncomms15888 (PMC5493763; doi:10.1038/ncomms15888)
Supplement: Supplementary Information — Supplementary Figures and Supplementary Tables [file ncomms15888-s1.pdf]

Type of file: pdf
Size of file: 0 KB
Title of file for HTML: Supplementary Information
Description: Supplementary Figures and Supplementary Tables

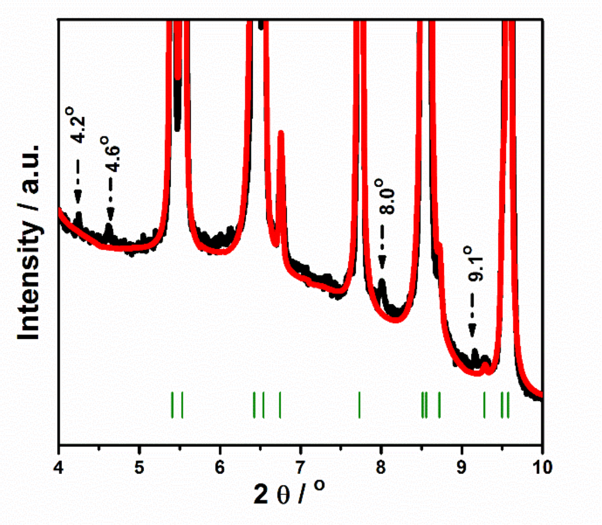

Supplementary Figure 1. Megascopic synchrotron diffraction pattern of $Na_2VTi(PO_4)_3$@C.

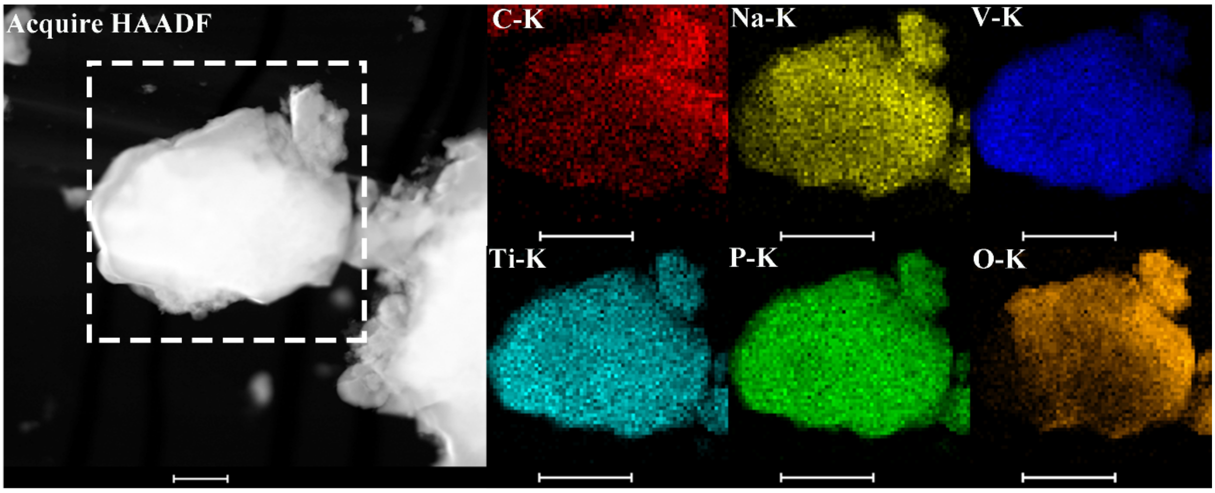

Supplementary Figure 2. HAADF-STEM images and mapping of $Na_2VTi(PO_4)_3$@C (Scale bar of HAADF and element mapping are 500 nm and 1 um, respectively).

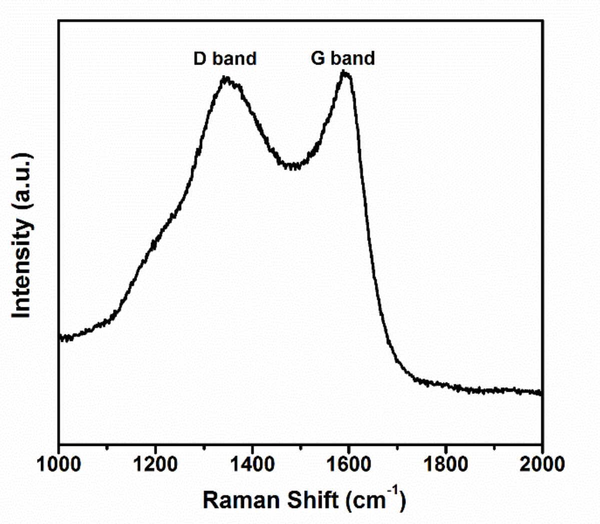

Supplementary Figure 3. Raman spectrum of $Na_2VTi(PO_4)_3$@C.

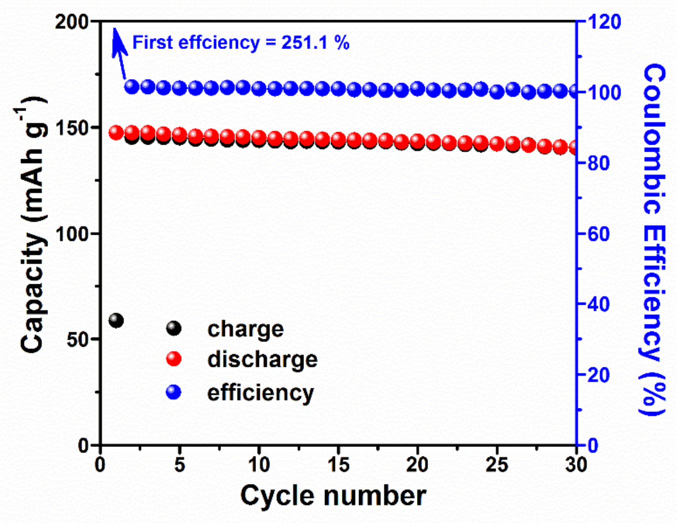

Supplementary Figure 4. The cycle performance and coulombic efficiency for 30 cycles.

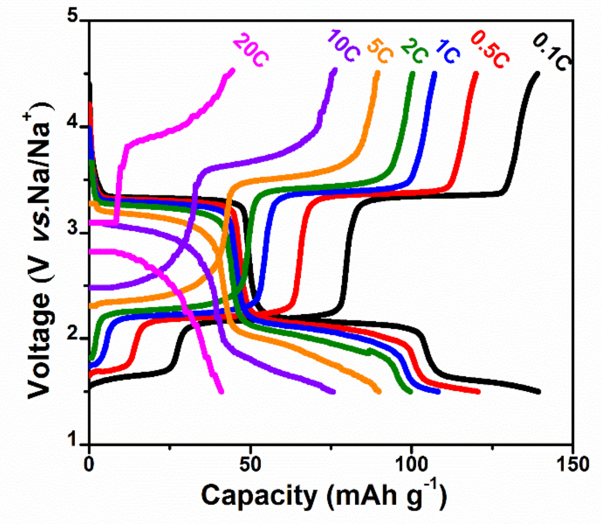

Supplementary Figure 5. Galvanostatic charge-discharge profiles at various current rates.

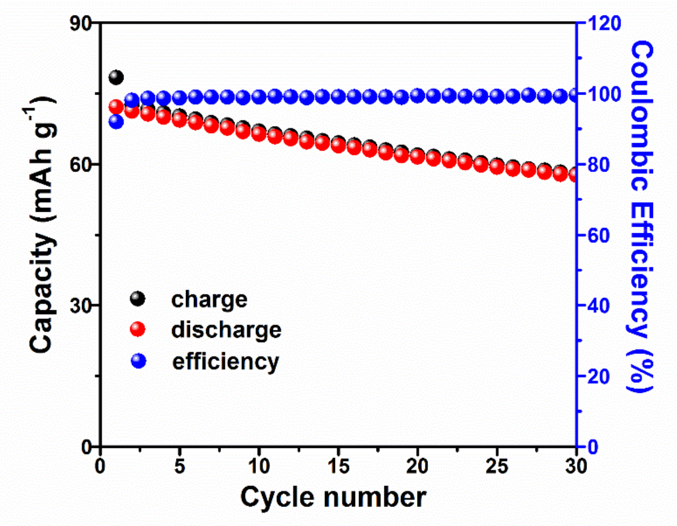

Supplementary Figure 6. The cycle performance and coulombic efficiency of $Na_2VTi(PO_4)_3$@C-based symmetrical full cell for 30 cycles at a curve rate of 1C between 0.5 and 2.0V.

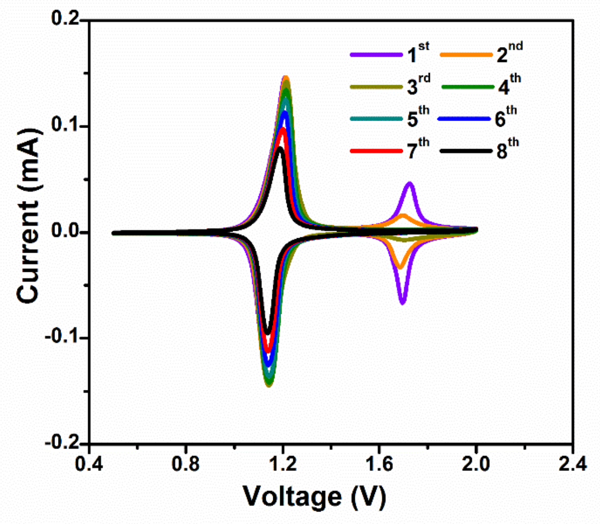

Supplementary Figure 7. Cyclic voltammograms at a scan rate of 0.1 mV s^{-1} between 0.5 and 2.0 V of the symmetric cell.

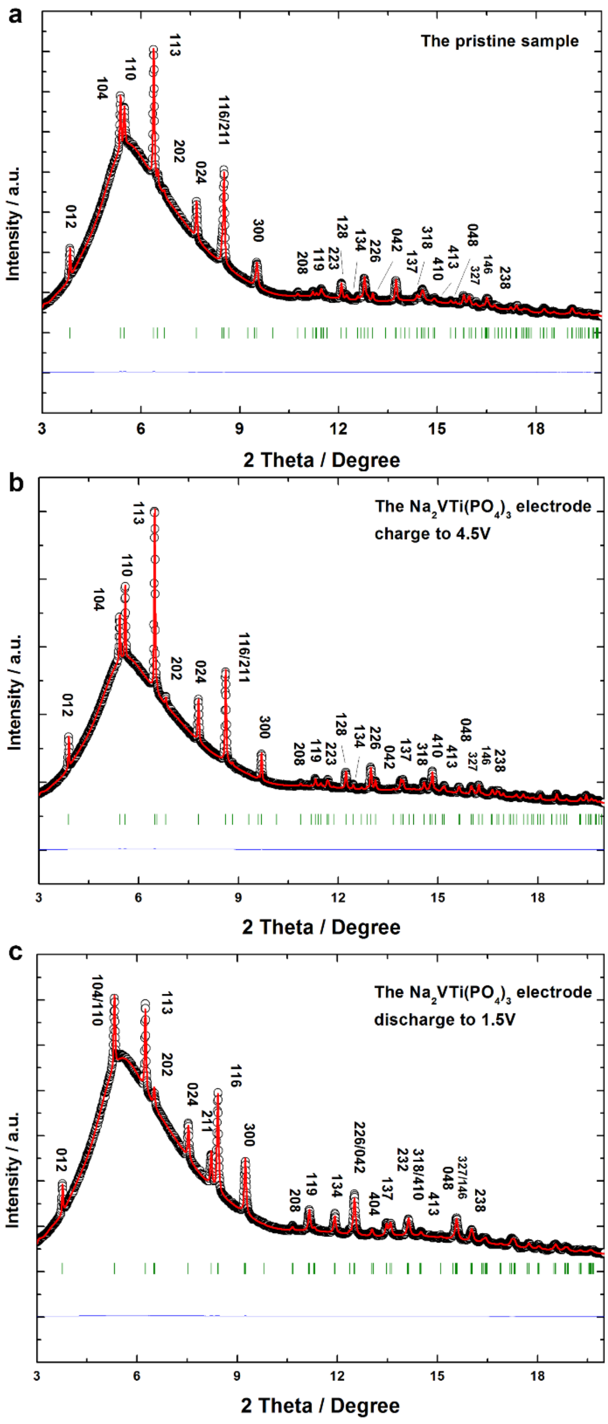

Supplementary Figure 8. Structure evolution of $Na_xVTi(PO_4)_3$ upon Na extraction/insertion: Rietveld refinement of the pristine $Na_2VTi(PO_4)_3$ (a), the $Na_2VTi(PO_4)_3$ electrode charge to 4.5 V (b) and the $Na_2VTi(PO_4)_3$ electrode discharge to 1.5 V (c). Black circles and red and blue lines represent the observed, calculated, and difference patterns, respectively. The green tick marks correspond to the Bragg reflections.

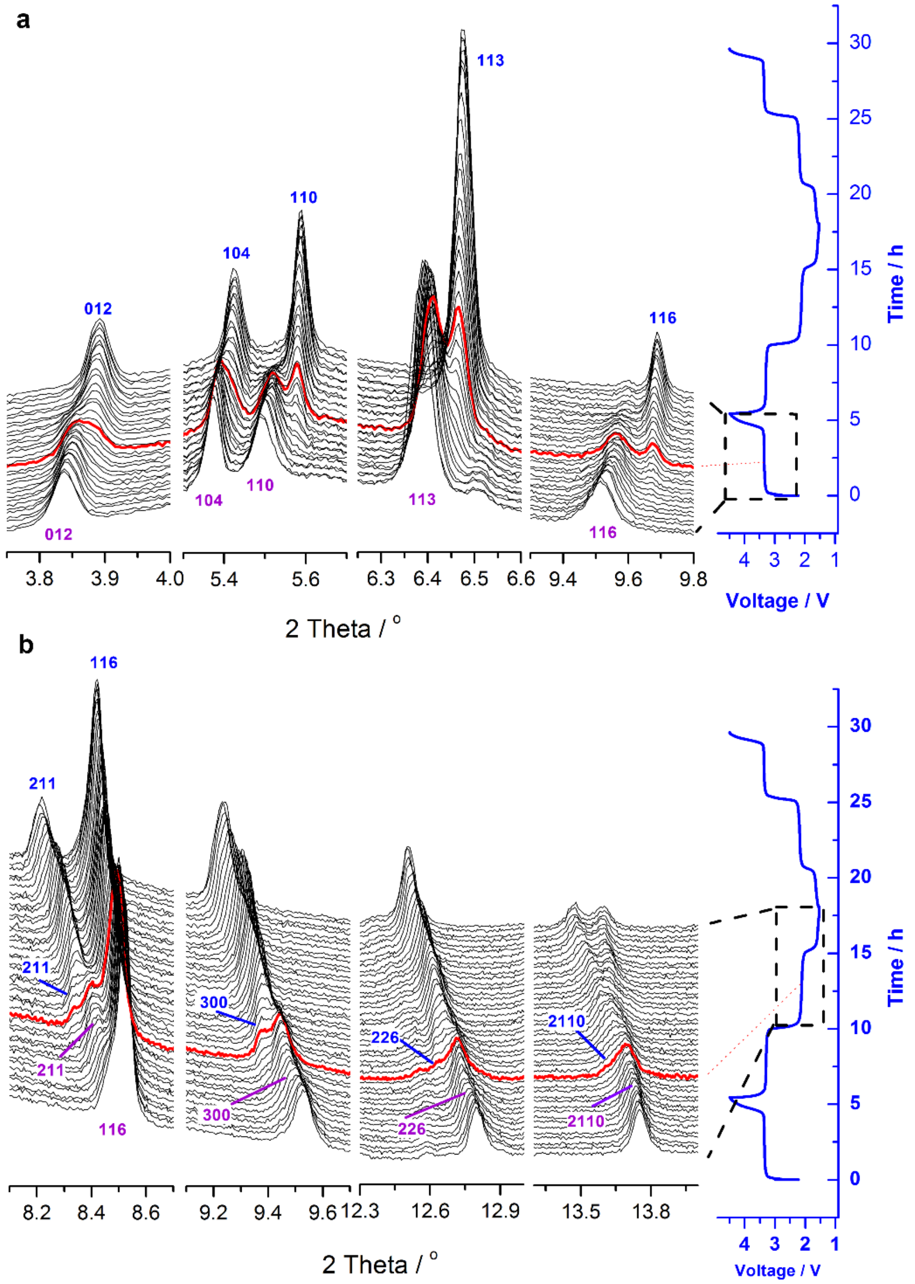

Supplementary Figure 9. **Enlarged *In situ* synchrotron XRD patterns.** (a) the enlarged electrochemical *in situ* synchrotron XRD patterns collected during the $Na_2VTi(PO_4)_3$ electrodes charge from 2.3 V to 4.5 V and (b) the enlarged electrochemical *in situ* synchrotron XRD patterns collected during the $Na_2VTi(PO_4)_3$ electrodes discharge from 3.3 V to 1.5 V.

Supplementary Table 1. The atomic positions of $Na_2VTi(PO_4)_3$@C nanocomposite upon Rietveld refinement results.

	Wyckoff position	x	y	z	Uiso	Occ
Na1	*6b*	0	0	0	0.0725(2)	1.028(6)
Na2	*18e*	0.6408(8)	0	0.25	0.042(3)	0.317(3)
Ti	*12c*	0	0	0.14642(4)	0.0028(2)	0.5
V	*12c*	0	0	0.14642(4)	0.0028(2)	0.5
P	*18e*	0.28765(1)	0	0.25	0.0159(4)	1
O1	*36f*	0.1727(2)	-0.0274(3)	0.19153(8)	0.0194(7)	1
O2	*36f*	0.1883(2)	0.1622(2)	0.08908(1)	0.0156(6)	1

Space Group: *R*-3*c*, *a*=8.59922(1), *c*=21.8181(4), R_{wp}=4.39, R_p=3.22

Supplementary Table 2. Comparison of sodium storage performance in the state-of-the-art full Na-ion batteries

Cathode || Anode	Capacity ($mA\ h\ g^{-1}$)	Voltage (V)	Capacity retention	Rate performance	Ref.
**$Na_3V_2(PO_4)_3$ || $NaTi_2(PO_4)_3$**	110 $mA\ h\ g^{-1}$ under current density of 13.3 $mA\ g^{-1}$	1.2	80% after 1000 cycles	90 $mA\ h\ g^{-1}$ under current density of 6.65A g^{-1}	13
**$Na_3V_2(PO_4)_3$ || Sb/C**	500 $mA\ h\ g^{-1}$ under current density of 0.1 A g^{-1}	2.1	70% after 100 cycles	-	19
**$Na_3V_2(PO_4)_3$ || CoS**	400 $mA\ h\ g^{-1}$ under current density of 0.5 A g^{-1}	2.2	68% after 100 cycles	-	20
**$Na_{7/9}Cu_{2/9}Fe_{1/9}Mn_{2/3}O_2$|| hard carbon**	350 $mA\ h\ g^{-1}$ under current density of 20 $mA\ g^{-1}$	2.5	81% after 50 cycles	-	21
**$Na_3Ni_2SbO_6$ || Sb/C**	110 $mA\ h\ g^{-1}$ under current density of 20 $mA\ g^{-1}$	3.0	68% after 50 cycles	-	22
**$NaNi_{0.5}Mn_{0.5}O_2$|| hard carbon**	250 $mA\ h\ g^{-1}$ under current density of 25 $mA\ g^{-1}$	3.0	44% after 80 cycles	-	34
**$Na_3V_2(PO_4)_3$|| $Na_{0.66}[Li_{0.22}Ti_{0.78}]O_2$**	95 $mA\ h\ g^{-1}$ under current density of 10.6 $mA\ g^{-1}$	2.6	67% after 20 cycles	75 $mA\ h\ g^{-1}$ under current density of 212 $mA\ g^{-1}$	35

**Symmetric $Na_{0.6}[Cr_{0.6}Ti_{0.4}]O_2$**	80 mA h g^{-1} under current density of 76 mA g^{-1}	2.5	78% after 100 cycles	55 mA h g^{-1} under current density of 912 mA g^{-1}	16
**Symmetric $Na_{0.8}Ni_{0.4}Ti_{0.6}O_2$**	80 mA h g^{-1} under current density of 20 mA g^{-1}	2.8	70% after 150 cycles	53 mA h g^{-1} under current density of 100 mA g^{-1}	17
**Symmetric $Na_{2.55}V_6O_{16}\cdot 0.6H_2O$**	130 mA h g^{-1} at 50 mA g^{-1}	1.5	85% after 100 cycles	-	30
**Symmetric $Na_{0.66}Ni_{0.17}Co_{0.17}Ti_{0.66}O_2$**	100 mA h g^{-1} under current density of 20 mA g^{-1}	3.1	76% after 1000 cycles	40 mA h g^{-1} under current density of 1.5 A g^{-1}	31
**Symmetric $NaNi_{0.33}Li_{0.11}Ti_{0.56}O_2$**	80 mA h g^{-1} under current density of 20 mA g^{-1}	2.3	63% after 100 cycles	40 mA h g^{-1} under current density of 100 mA g^{-1}	32
**Symmetric $Na_3V_2(PO_4)_3$**	100 mA h g^{-1} under current density of 58.8 mA g^{-1}	1.7	75% after 200 cycles	40 mA h g^{-1} under current density of 1.176 A g^{-1}	18
**Symmetric $Na_3V_2(PO_4)_3$**	100 mA h g^{-1} under current density of 23 mA g^{-1}	1.7	71% after 280 cycles	30 mA h g^{-1} under current density of 1.176 A g^{-1}	33
**Symmetric $Na_3Ti_2(PO_4)_3$**	-	1.7	-	-	15
**Symmetric $Na_2VTi(PO_4)_3$**	80 mA h g^{-1} under current density of 125 mA g^{-1}	1.2	74% after 10000 cycles	49 mA h g^{-1} under current density of 2.5 A g^{-1}	This work

Supplementary Table 3 Structural parameters of the pristine $Na_2VTi(PO_4)_3$@C electrodes which were obtained from *in situ* synchrotron X-ray diffraction Rietveld analysis.

S.G. *R*-3*c* *a*=8.6170(3) Å, *c*=21.8127(8) Å						
	Wyckoff position	x	y	z	Uiso	Occ
Na1	*6b*	0	0	0	0.083(4)	1
Na2	*18e*	0.6460(2)	0	0.25	0.069(1)	0.353(1)
Ti	*12c*	0	0	0.14682(1)	0.0033(8)	0.5
V	*12c*	0	0	0.14682(1)	0.0033(8)	0.5
P	*18e*	0.2891(4)	0	0.25	0.0332(2)	1
O1	*36f*	0.1759(6)	-0.0215(7)	0.1919(2)	0.038(2)	1
O2	*36f*	0.1854(5)	0.1588(5)	0.0882(2)	0.0166(2)	1
R_{wp}=0.78, R_p=0.6						

Supplementary Table 4 Structural parameters of the $Na_2VTi(PO_4)_3$@C electrodes charge to 4.5V which were obtained from *in situ* synchrotron X-ray diffraction Rietveld analysis.

S.G. *R*-3*c* *a*=8.4650(2) Å, *c*=21.6886(7) Å						
	Wyckoff position	x	y	z	Uiso	Occ
Na1	*6b*	0	0	0	0.034(3)	1.008(1)
Na2	*18e*	0.623(2)	0	0.25	0.01	0.023(4)
Ti	*12c*	0	0	0.14504(1)	0.0058(7)	0.5
V	*12c*	0	0	0.14504(1)	0.0058(7)	0.5
P	*18e*	0.2860(3)	0	0.25	0.0153(1)	1
O1	*36f*	0.1707(5)	-0.0262(6)	0.1904(2)	0.0223(2)	1
O2	*36f*	0.1875(5)	0.1589(5)	0.0893(3)	0.0083(1)	1
R_{wp}=0.8, R_p=0.62						

Supplementary Table 5 Structural parameters of the $Na_2VTi(PO_4)_3$@C electrodes discharge to 1.5V which were obtained from *in situ* synchrotron X-ray diffraction Rietveld analysis.

S.G. *R*-3*c* *a*=8.8770(3) Å, *c*=21.8152(1) Å						
	Wyckoff position	x	y	z	Uiso	Occ
Na1	*6b*	0	0	0	0.049(9)	0.82(3)
Na2	*18e*	0.6428(1)	0	0.25	0.018(5)	0.838(1)
Ti	*12c*	0	0	0.14752(1)	0.0060(1)	0.5
V	*12c*	0	0	0.14752(1)	0.0060(1)	0.5
P	*18e*	0.2921(6)	0	0.25	0.0239(2)	1
O1	*36f*	0.1906(9)	-0.0298(9)	0.1900(3)	0.008(3)	1
O2	*36f*	0.1965(1)	0.1678(1)	0.0947(5)	0.015(2)	1
R_{wp}=0.89, R_p=0.68						
